# Antidepressant treatment is associated with changes in early maladaptive schemas in major depressive disorder: a prospective study

**DOI:** 10.3389/fpsyt.2026.1865222

**Published:** 2026-08-14

**Authors:** Ahlem Hajri, Abdennour Karmous, Sirine Laabidi, Emira Khelifa, Mohamed Hajri, Tarak Dhaouadi, Haifa Zalila, Amira Maamri

**Affiliations:** 1 Outpatient and Emergency Department, Razi Hospital, Manouba, Tunisia; 2 University of Tunis El Manar, Tunis, Tunisia; 3 Department of Visceral and Digestive Surgery, Mongi Slim Hospital, La Marsa, Tunisia; 4 Research Laboratory in Immunology of Renal Transplantation and Immunopathology (LR03SP01), Charles Nicolle Hospital, Tunis El Manar University, Tunis, Tunisia

**Keywords:** early maladaptive schemas, depressive disorder, depression, antidepressants, effects

## Abstract

**Introduction:**

Early maladaptive schemas (EMS) have been increasingly recognized as key cognitive vulnerability factors in depression. This study aimed to evaluate the effects of antidepressant treatment on early maladaptive schemas (EMS) and to examine the relationships between EMS and demographic and clinical factors in major depression.

**Methods:**

We conducted a three-month prospective study including 80 patients diagnosed with a major depressive disorder. EMS were assessed using the Young Schema Questionnaire Short Form (YSQ-S2), and depression severity was measured using the Hamilton Depression Rating Scale at baseline (T0) and after 3 months of antidepressant treatment (T3).

**Results:**

After 3 months of antidepressant treatment, mean EMS scores significantly decreased, except for the *Entitlement* schema. No significant correlations were found between EMS scores and depression severity at baseline or follow-up. Additionally, baseline depression severity did not predict changes in EMS scores, except for the *defectiveness/shame* schema.

**Conclusion:**

Antidepressant treatment was associated with a significant reduction in most EMS. These findings underscore the importance of additional research on cognitive plasticity of EMS and the mechanisms underlying treatment effects, which could inform more personalized and effective therapeutic strategies.

## Introduction

Cognitive theories occupy a central place among psychological approaches to depression, providing an essential framework for its clinical understanding. Within this perspective, the concept of schemas has played a major role in cognitive models of psychopathology since their development. According to Beck’s cognitive theory of depression (1), negative self-schemas represent key vulnerability factors for several psychological disorders, including depression.

An early maladaptive schema (EMS) is defined by Young as “a broad, pervasive theme regarding oneself and one’s relationships with others, developed during childhood and elaborated throughout life, and dysfunctional to a significant degree” (2). These EMS constitute an individual’s core beliefs about the self and the world. They are formed during childhood and adolescence in response to adverse experiences or unmet psychological needs (2). These schemas then function as templates for processing subsequent experiences, which in turn reinforce and maintain them (3, 4). According to Young’s model, EMS represent a cognitive vulnerability to depressive disorders and play a role in both the onset and course of depression (2). They also constitute the theoretical foundation for a growing number of schema-focused therapies.

Although Young conceptualized early maladaptive schemas (EMS) as stable and enduring structures, their invariance over time remains debated. Riso et al. reported moderate to good long-term stability in depressed patients over a 2.5- to 5-year period (5). However, growing evidence suggests that EMS are not entirely rigid. Affective fluctuations, particularly during depressive episodes, may temporarily activate or intensify negative schemas, thereby influencing individuals’ perceptions of themselves, others, and the world (6–9). In addition, therapeutic interventions including pharmacological treatments (10) and cognitive-behavioral therapy (11) may contribute to changes in these schemas.

In this context, EMS may be better understood as partially dynamic constructs, exhibiting a certain degree of cognitive plasticity. However, it remains unclear whether observed changes reflect direct modification of underlying cognitive structures or secondary effects of mood improvement.

This potential plasticity highlights the importance of investigating how therapeutic interventions, including antidepressant pharmacological treatment, may modulate these cognitive structures. To our knowledge, no Tunisian study has examined early maladaptive schemas in relation to major depressive disorder or the potential impact of pharmacological treatment.

In this context, the main objective of our study was to assess within-person changes in EMS scores during three months of antidepressant treatment. Secondary objectives were to examine the associations between EMS and clinical or course characteristics, as well as depressive severity.

## Materials and methods

### Study design

We conducted a prospective, uncontrolled, pre-post observational study with a three-month follow-up.

Patients were recruited over a ten-month period, between February 2023 and December 2023. Patients who met the inclusion criteria were asked to complete two standardized questionnaires. Other data were collected from patients’ medical records using standardized form.

The scales were administered in two stages in order to compare scores before and after antidepressant treatment:

-at T0: during the first consultation. Baseline (T0) assessments were performed immediately before the initiation of antidepressant treatment. Consequently, all participants were antidepressant-naïve at the time of the baseline evaluation.

-at T3: after 3 months of antidepressant treatment. A minimum duration of 6 to 8 weeks is recommended to assess the initial response to antidepressant treatment. We set the three-month period to allow for possible therapeutic adjustment with a view to achieving an optimal response.

### Study population

#### Inclusion criteria

We included patients aged between 18 and 65, who had consulted for the first time at the outpatient department of Razi Hospital in 2023 for a major depressive episode according to the criteria of the Diagnostic and Statistical Manual DSM-V and who had been initiated on antidepressant treatment during this first consultation. The patients included did not receive cognitive or EMS-focused psychotherapy during the study period, after verification of their medical records, in order to avoid a confounding factor in the evaluation of the specific effect of antidepressant treatment on these schemas. However, in cases of insufficient clinical response, they were eligible for specialized cognitive psychotherapy at the end of the evaluation period.

#### Exclusion criteria

The exclusion criteria were as follows: patients who had already received antidepressant treatment before the baseline assessment (T0), patients with intellectual impairment or dementia that could hinder communication or the administration of assessment scales; patients with depressive disorders secondary to a somatic medical condition; depressive disorders induced by psychoactive substances or medications; a personal history of bipolar disorder; or chronic psychotic disorders.

Additionally, patients who withdrew their consent during the study or discontinued outpatient follow-up or antidepressant treatment before study completion were excluded.

Participants were recruited consecutively during the study period. No formal a priori sample size calculation was performed due to the exploratory nature of the study and the lack of prior local data on expected changes in early maladaptive schema scores following antidepressant treatment.

### Data and variables studied

#### Socio-demographic data

Age, gender, marital status, level of education, employment status.

#### Clinical and course characteristics of depressive disorder

Age of onset and duration of major depressive disorder, severity of current depressive episode, characteristics of current depressive episode (melancholic, psychotic, mixed, atypical), course type (isolated episode, recurrent, persistent), current treatment, clinical evolution at 12 weeks of antidepressant treatment. (full or partial remission, or no remission).

### Assessment tools

Socio-demographic and clinical data were collected from medical records and clinical interviews.

To assess EMS and the severity of depression, we used the short version of the Young Schema Questionnaire (YSQ-S2) and the Hamilton Depression Rating Scale (HDRS), respectively.

#### Young schema questionnaire (YSQ-S2)

EMS were assessed using the short version of the Young Schema Questionnaire (YSQ-S2) validated in Arabic (12). This questionnaire was developed by Young and Brown in 1994 (2). It is available in two versions, long and short. The short version (75 items), has been shown to produce results equivalent to the long version (205 items) (13).

YSQ-S2 is a self-administered questionnaire consisting of 75 items and measuring 15 maladaptive early schemas: emotional deprivation, abandonment/instability, mistrust/abuse, social isolation, defectiveness/shame, failure, dependence/incompetence, vulnerability, enmeshment, subjugation, self-sacrifice, emotional inhibition, unrelenting standards, entitlement, insufficient self-control. A detailed description of each of the 15 schemas and of the scoring procedure is provided in **Box 1**.

#### Hamilton Depression Rating Scale

The Hamilton Depression Rating Scale (HDRS) is a clinician-rated scale consisting of 17 items used to assess the severity of a depressive episode. We used the validated French language version (14).

This scale has 17 items, each rated from 0 to 2 or from 0 to 4. The total score ranges from 0 to 52.

According to standard thresholds, HDRS scores were interpreted as follows: ≤7 indicates no depression, 8–17 mild depression, 18–24 moderate depression, and ≥25 severe depression.

### Statistical analysis

In this study, Excel software was used to enter patient data.

#### Descriptive study

For qualitative variables, we calculated simple frequencies and relative frequencies (percentages). For quantitative variables, we determined extreme values, means, standard deviations, and medians.

#### Analytical study

Statistical analysis was performed using SPSS 27, with a significance threshold of 0.05. The normality of quantitative variables was assessed using histograms, asymmetry and kurtosis coefficients, as well as the Kolmogorov-Smirnov and Shapiro-Wilk tests. A variable was considered quasi-normal if these indices were between –1 and +1 and if the tests were not significant. Comparisons between groups for normal or quasi-normal variables were performed using ANOVA after checking for homogeneity of variances, while for non-normal variables, the Kruskal-Wallis test was used. In addition, differences in the change in EMS scores according to the type of antidepressant received were examined using one-way ANOVA, with the Kruskal–Wallis test for non-normally distributed variables.

To evaluate EMS scores before and after treatment, paired-samples t-tests were used for normally or approximately normally distributed variables, whereas the Friedman test was used for non-normally distributed variables. Effect sizes for paired comparisons were reported as Cohen’s d with 95% confidence intervals, using the square root of the average of the T0 and T3 variances as the standardizer, as implemented in IBM SPSS Statistics version 27.

Finally, correlations between quantitative variables were calculated using Pearson or Spearman tests depending on the normality of the distributions, with the effect size estimated from the r² coefficient for Pearson correlations.

To test whether schema change was dependent on mood-state change, change scores were computed between baseline (T0) and 3-month follow-up (T3) for the Hamilton Depression Rating Scale (ΔHDRS) and for each early maladaptive schema as well as the total EMS score (ΔEMS), defined as the difference (T0 − T3) so that positive values denote improvement. The association between ΔHDRS and each ΔEMS was then examined using Pearson and Spearman correlations and linear regression, with p-values corrected for multiple comparisons across the 15 schemas using the Benjamini–Hochberg false-discovery-rate (FDR) procedure.

## Results

A total of 80 patients were initially included. Of these, 4 patients did not complete the 3-month follow-up assessment and were considered lost to follow-up. The final sample included 76 participants who completed both assessments and were included in the longitudinal analysis.

### Descriptive analysis

#### Socio-demographic characteristics

**Table 1** summarizes the distribution of patients according to socio-demographic characteristics. The mean age of patients was 41.9 years with a standard deviation of 10.9 years and extreme ages of 19 and 64 years. Our patient group consisted of 21 men and 59 women, corresponding to a male/female sex ratio of 0.35.

**Table 1:** Socio-demographic characteristics of patients.

Characteristic	n (%)
Age (years)	41.9 ± 10.9
Sex-ratio (M/F)	0.35 (21/59)
Education level	
Primary	36 (45)
Secondary	26 (32.5)
University	18 (22.5)
Occupation	
Manual worker	26 (32.5)
Salaried employee	16 (20)
Self-employed professional	4 (5)
Student	6 (7.5)
Unemployed	28 (35)
Marital Status	
Married	43 (53.8)
Single	17 (21.3)
Divorced	12 (15)
Widowed	8 (10)
Socioeconomic status	
Low	50 (62.5)
Middle	30 (37.5)

#### Clinical characteristics

The mean age at the first depressive episode was 38.36 ± 11 years, with ages ranging from 18 to 62 years. Baseline depression severity, as measured by the HDRS, was mild in 41.25% of cases (33 patients), moderate in 40% of cases (32 patients), and severe in 18.75% of cases (15 patients). At the end of the follow-up period (3 months), 56.58% of patients had experienced partial remission of symptoms (n=43) and 42.1% of patients had experienced full remission of symptoms (n=32).

Regarding the type of depressive disorder, 38 patients had an isolated major depressive episode (47.5%), 19 patients (23.8%) had recurrent depressive disorder, and 23 patients met the criteria for persistent depressive disorder (28.7%).

Anxiety characteristics were observed in 30% of cases (24 patients), while mixed characteristics were found in 2.5% of cases (2 patients).

No patients in the study presented atypical, melancholic, or psychotic characteristics during the current depressive episode.

##### Treatment characteristics

All patients received antidepressant treatment. At treatment initiation, Fluoxetine was the most frequently prescribed drug (65%, n=51), followed by escitalopram (18.5%, n=15) and sertraline (15%, n=11), while two patients (2.5%) received paroxetine. All patients received the recommended minimum effective dose for the prescribed antidepressant (fluoxetine 20 mg/day, paroxetine 20 mg/day, sertraline 50 mg/day, escitalopram 10 mg/day). During follow-up, a dose increase was required in 27.2% of patients (n = 22). Among patients who required dose increase, the maximum prescribed doses were 40 mg/day for fluoxetine, 20 mg/day for escitalopram, 100 mg/day for sertraline, 20 mg/day for paroxetine. Treatment modifications were observed in 13 patients (16.25%). The most frequent switch was from fluoxetine to escitalopram (n = 4, 30.8%).

#### EMS scores at T0

The EMS with the highest scores at T0 were: self-sacrifice, social isolation, emotional deprivation, and abandonment/instability (**Table 2**).

**Table 2:** Scores for maladaptive early schemas in patients with depressive disorder.

EMS	Mean	SD
Emotional deprivation	22.42	5.70
Abandonment/instability	23.62	5.13
Mistrust/abuse	20.53	6.63
Social isolation	22.17	4.70
Defectiveness/Shame	11.45	5.37
Failure	14.91	6.71
Dependence/Incompetence	17.07	4.67
Vulnerability	21.76	5.95
Enmeshment	12.79	6.15
Subjugation	14.74	6.05
Self-sacrifice	24.03	4.61
Emotional inhibition	18,16	3.15
Unrelenting standards	18.49	4.71
Entitlement	17.26	5.88
Insufficient self-control	15.67	5.53
Total schemas scores	274.32	36.15

SD, Standard-Deviation.

### Analytical study

#### Change in EMS scores after 3 months of antidepressant treatment

Comparison of mean EMS scores at T0 and T3 showed a significant decrease in mean EMS scores, with the exception of the “entitlement “ schema, after 3 months of antidepressant treatment (**Table 3**). The observed changes were associated with effect sizes ranging from small to very large (Cohen’s d = 0.23–1.71). The largest effects were observed for the Emotional Deprivation (d = 1.71), Social Isolation (d = 1.45), and total EMS score (d = 1.23), whereas the Entitlement schema showed a negligible effect size (d = −0.03).

**Table 3:** Change in EMS scores after 3 months of antidepressant treatment.

EMS	T0	T3	p	Effect size (Cohen’s d, 95% CI)
Emotional deprivation	22.42 ± 5.70	13.83 ± 4.26	<0.001	1.71[1.35, 2.06]
Abandonment/instability	23.62 ± 5.13	21.5 ± 4.79	<0.001	0.43[0.19, 0.66]
Mistrust/abuse	20.53 ± 6.63	15.3 ± 4.18	<0.001	0.94[0.67, 1.21]
Social Isolation	22.17 ± 4.70	16.34 ± 3.17	<0.001	1.45[1.13, 1.77]
Defectiveness/shame	11.45 ± 5.37	10.14 ± 4.88	0.039	0.25[0.03, 0.48]
Failure	14.91 ± 6.71	12.79 ± 5.76	<0.001	0.34[0.11, 0.57]
Dependence/incompetence	17.07 ± 4.67	13.76 ± 3.73	<0.001	0.78[0.52, 1.04]
Vulnerability	21.76 ± 5.95	16.93 ± 4.63	<0.001	0.91[0.64, 1.17]
Enmeshment	12.79 ± 6.15	10.57 ± 5.18	<0.001	0.39[0.16, 0.62]
Subjugation	14.74 ± 6.05	13.42 ± 5.64	<0.001	0.23[-0.004, 0.45]
Self-sacrifice	24.03 ± 4.61	20.78 ± 4.25	<0.001	0.73[0.48, 0.98]
Emotional inhibition	18.16 ± 3.14	16.05 ± 3.01	<0.001	0.68[0.43, 0.93]
Unrelenting standards	18.49 ± 4.71	17.13 ± 4.19	<0.001	0.30[0.07, 0.53]
Entitlement	16.88 ± 5.41	17.26 ± 5.88	0.825	-0.03[-0.26, 0.19]
Insufficient self-control	15.67 ± 5.53	14.37 ± 4.96	<0.001	0.25[0.02, 0.48]
Total score	274.32 ± 36.15	230.13 ± 35.68	<0.001	1.23[0.93, 1.53]

CI, Confidence Interval.

A linear regression showed that baseline depression severity (Hamilton T0) did not predict the change in EMS scores except for the Defectiveness/Shame schema (**Table 4**). Although the Defectiveness/Shame schema was nominally associated with changes in EMS scores (unadjusted p = 0.03), this association did not remain significant after Benjamini–Hochberg correction for multiple comparisons.

**Table 4:** Linear regression of ΔEMS scores on depression severity (at T0).

EMS	Coefficient(B)	95% CI	SE	t	p
Emotional deprivation	0.09	[-0.15, 0.34]	0.12	0.78	0.44
Abandonment/instability	-0.08	[-0.25, 0.08]	0.08	-0.97	0.33
Mistrust/abuse	0.04	[-0.11, 0.19]	0.07	0.54	0.58
Social isolation	-0.02	[-0.19, 0.14]	0.08	-0.31	0.75
Defectiveness/Shame	-0.25	[-0.49, -0.01]	0.12	-2.10	**0.03**
Failure	-0.06	[-0.13, 0.01]	0.03	-1.76	0.08
Dependence/incompetence	0.02	[-0.02, 0.06]	0.02	0.84	0.40
Vulnerability	0.02	[-0.06, 0.12]	0.04	0.60	0.55
Enmeshment	0.01	[-0.03, 0.06]	0.02	0.49	0.62
Subjugation	-0.044	[-0.12, 0.04]	0.04	-1.03	0.30
Self-sacrifice	0.02	[-0.07, 0.11]	0.04	0.38	0.70
Emotional inhibition	0.03	[-0.04, 0.11]	0.04	0.81	0.41
Unrelenting standards	0.01	[-0.04, 0.06]	0.02	0.36	0.71
Entitlement	-0.14	[-0.82, 0.55]	0.34	-0.39	0.69
Insufficient self-control	0.03	[-0.03, 0.09]	0.03	0.99	0.32
Total Score	-0.30	[-1.14, 0.53]	0.42	-0.73	0.46

Bold values indicate statistically significant associations at p < 0.05 (uncorrected for multiple comparisons).

Moreover, the reduction in the total EMS score did not differ significantly according to the specific antidepressant received (fluoxetine: 43.2 ± 21.1; escitalopram: 45.4 ± 11.0; sertraline: 45.9 ± 11.4; one-way ANOVA F = 0.14, p = 0.87; Kruskal–Wallis p = 0.94; η² = 0.004), and the magnitude of the antidepressant response (ΔHDRS) was comparable across drugs (p = 0.57).

#### EMS and clinical characteristics

We did not find any statistically significant relationships between the mean EMS scores and the age of onset of depressive disorder, or between the mean EMS scores and the specifications (mixed, psychotic, anxious characteristics).

However, patients with a history of suicide attempts had significantly higher scores for the following EMS: defectiveness/shame (p=0.003), unrelenting standards (p=0.023), entitlement (p=0.042), Insufficient self-control (p=0.016), and total score (p=0.041).

#### EMS and Hamilton scale scores

We found no significant correlations between the scores on the various EMS means and the initial severity of the depressive disorder or the severity of the depressive disorder after 3 months of treatment according to the Hamilton scale scores (**Table 5**).

**Table 5:** Correlations between EMS scores and depression severity.

EMS	HDRS* T0 scores			HDRS* T3 scores		
	r	95%CI	p	r	95%CI	p
Emotional deprivation	-0.06	[-0.28, 0.15]	0.56	-0.1	[-0.32, 0.13]	0.39
Abandonment/Instability	-0.03	[-0.25, 0.18]	0.76	0.16	[-0.07, 0.37]	0.18
Mistrust/abuse	0.06	[-0.15, 0.28]	0.56	0.14	[-0.09, 0.35]	0.23
Social isolation	-0.04	[-0.26, 0.17]	0.66	0.03	[-0.19, 0.25]	0.78
Defectiveness/Shame	-0.11	[-0.32, 0.11]	0.32	0.11	[-0.11, 0.33]	0.32
Failure	-0.17	[-0.37, 0.05]	0.13	-0.14	[-0.36, 0.08]	0.21
Dependence/Incompetence	-0.03	[-0.25, 0.18]	0.75	0.03	[-0.25, 0.19]	0.80
Vulnerability	0.12	[-0.10, 0.33]	0.28	0.14	[-0.08, 0.36]	0.22
Enmeshment	0.01	[-0.20, 0.23]	0.9	0.04	[-0.19, 0.26]	0.75
Subjugation	-0.13	[-0.34, 0.09]	0.24	-0.07	[-0.29, 0.15]	0.51
Self-sacrifice	0.03	[-0.18, 0.25]	0.77	0.04	[-0.19, 0.26]	0.75
Emotional inhibition	0.13	[-0.08, 0.34]	0.23	0.05	[-0.17, 0.28]	0.63
Unrelenting standards	0.12	[-0.09, 0.33]	0.27	0.09	[-0.13, 0.31]	0.42
Entitlement	0.12	[- 0.10, 0.33]	0.28	0.09	[-0.13, 0.31]	0.41
Insufficient self-control	0.13	[- 0.09, 0.34]	0.25	0.06	[-0.17, 0.28]	0.60
**Total score**	0.01	[-0.20, 0.23]	0.92	0.10	[-0.12, 0.32]	0.36

*HDRS, Hamilton Depression Rating Scale.

r = Pearson correlation coefficient; p = two-tailed significance value.

Bold values indicate statistically significant associations at p < 0.05 (uncorrected for multiple comparisons).

To further test whether the reduction in schema scores merely reflected the concurrent improvement in mood, we examined the relationship between the individual change scores. Although both depressive symptoms (HDRS: 18.6 ± 5.0 to 8.6 ± 2.4; p < 0.001) and the total EMS score (274.4 ± 36.2 to 230.1 ± 35.7; p < 0.001) decreased significantly, the magnitude of depressive improvement (ΔHDRS) was not associated with the magnitude of schema reduction (ΔEMS), either for the total EMS score (r = −0.06, 95% CI [−0.28, 0.17], p = 0.60) or for any individual schema after correction for multiple comparisons (all p (FDR) ≥ 0.43; **Table 6**). Only the Defectiveness/Shame schema reached nominal significance (r = −0.25, p = 0.03), in the direction opposite to that predicted by mood-state dependency, but it did not survive Benjamini–Hochberg correction.

**Table 6:** Correlations between the change in depression severity (ΔHDRS) and the change in early maladaptive schema scores (ΔEMS) after 3 months of antidepressant treatment.

EMS	Δ HDRS* scores			
	*r*	95% CI	*p*	*p (FDR)*
Emotional deprivation	0.10	[-0.13, 0.32]	0.39	0.70
Abandonment/Instability	-0.13	[-0.35, 0.10]	0.25	0.70
Mistrust/abuse	0.07	[-0.16, 0.29]	0.54	0.70
Social isolation	-0.04	[-0.26, 0.19]	0.76	0.76
Defectiveness/Shame	-0.25	[-0.45, -0.03]	0.03	0.43
Failure	-0.19	[-0.40, 0.04]	0.10	0.70
Dependence/Incompetence	0.13	[-0.10, 0.34]	0.27	0.70
Vulnerability	0.10	[-0.13, 0.32]	0.39	0.70
Enmeshment	0.06	[-0.17, 0.28]	0.63	0.72
Subjugation	-0.08	[-0.30, 0.15]	0.51	0.70
Self-sacrifice	0.08	[-0.15, 0.30]	0.48	0.70
Emotional inhibition	0.10	[-0.13, 0.32]	0.40	0.70
Unrelenting standards	0.07	[-0.16, 0.29]	0.56	0.70
Entitlement	-0.03	[-0.26, 0.19]	0.76	0.76
Insufficient self-control	0.14	[-0.09, 0.35]	0.23	0.70
**Total score**	**-0.06**	**[-0.28, 0.17]**	**0.60**	**—**

*HDRS, Hamilton Depression Rating Scale.

Δ (change score) = score at T0 − score at T3; positive values indicate improvement (reduction of symptom severity or schema intensity), n = 76.

r = Pearson correlation coefficient; p = two-tailed significance value; p (FDR) = Benjamini–Hochberg-corrected p-value across the 15 schemas.

No correlation remained significant after FDR correction (all p (FDR) ≥ 0.43). Spearman rank correlations yielded concordant results.

Bold values indicate statistically significant associations at p < 0.05 (uncorrected for multiple comparisons).

#### EMS and remission of depressive disorder

Patients who achieved partial remission, full remission, or no remission over the 3-month period were comparable in terms of mean EMS scores at T0.

#### EMS and course type of depressive disorder

We did not find any significant differences between the scores of the various EMS based on the course type of depressive disorder (**Table 7**).

**Table 7:** EMS and course type of depressive disorder.

EMS	Isolated major depressive disorder (n=38)	Recurrent depressive disorder (n=19)	Persistent depressive disorder (n=23)	p
Emotional deprivation	21.87 ± 5.24	19.05 ± 7.89	23.74 ± 6.01	0.55
Abandonment/instability	22.58 ± 5.28	22.05 ± 5.60	25.17 ± 4.94	0.1
Mistrust/abuse	20.05 ± 6.29	18.84 ± 7.66	21.09 ± 7.26	0.58
Social isolation	22 ± 4.82	22 ± 5.22	21.78 ± 4.85	0.98
Defectiveness/Shame	10.95 ± 5.08	11.26 ± 6.24	11.78 ± 5.16	0.84
Failure	14.87 ± 6.38	12.37 ± 6.95	17.09 ± 6.41	0.07
Dependence/incompetence	16.47 ± 3.91	16 ± 5.85	18.39 ± 4.75	0.19
Vulnerability	20.68 ± 6.32	21.21 ± 5.80	23.17 ± 5.19	0.27
Enmeshment	12.32 ± 5.99	12.05 ± 4.61	13.35 ± 7.4	0.75
Subjugation	14.39 ± 5.9	12.58 ± 6.48	16.26 ± 5.76	0.14
Self-sacrifice	23.84 ± 4.09	23.32 ± 5.29	23.91 ± 5.28	0.9
Emotional inhibition	18.34 ± 2.86	18.21 ± 3.52	17.74 ± 3.44	0.77
Unrelenting standards	18.34 ± 4.62	18.37 ± 5.16	18.83 ± 4.45	0.92
Entitlement	17.24 ± 5.8	17.05 ± 5.13	15.83 ± 4.97	0.6
Insufficient self-control	15.55 ± 5.29	16.21 ± 5.79	15.3 ± 5.56	0.86

## Discussion

### Main results

The EMS with the highest scores in subjects with major depressive disorder were: self-sacrifice, emotional deprivation, and abandonment/instability.

We found no significant correlations between average EMS scores and the severity of depression, its course type, or the type of remission.

After three months of antidepressant treatment, average EMS scores decreased significantly, with the exception of the entitlement schema. The observed changes were associated with small to very large effect sizes (Cohen’s d = 0.23–1.71). The largest effects were observed for the total EMS score and the Emotional Deprivation and Social Isolation schemas, whereas the Entitlement schema showed a negligible effect size (d = −0.03).

A linear regression showed that baseline depression severity (Hamilton T0) did not predict the change in EMS scores except for the Defectiveness/Shame schema.

### EMS in depression

In our study, the EMS with the highest scores were: self-sacrifice, social isolation, emotional deprivation, and abandonment/instability. However, in the absence of a control group, it is not possible to determine whether these EMS are specifically related to depressive disorder.

Our findings are consistent with data from a meta-analysis conducted by Thimm and Chang, 2022, which analyzed EMS links with a wide range of psychiatric disorders, including case-control studies. A total of eight studies examined EMS in depressed patients. The results indicated that the largest effect sizes were for social isolation, abandonment/instability, emotional deprivation, defectiveness/shame and negativity/pessimism (15).

In another Australian systematic review and meta-analysis conducted by Bishop et al. in 2021, which included 51 studies (49 cross-sectional studies and 2 longitudinal studies) with sample sizes ranging from 38 to 1,529 subjects, significant positive correlations with depression were reported across the entire spectrum of EMS with varying effect sizes (small to large). Large pooled effect size estimates were found between depression and schemas of defectiveness/shame and social isolation, while moderate pooled effect sizes were found between depression and EMS of emotional deprivation, abandonment/instability, mistrust/abuse, and failure (16).

The relatively strong associations between these EMS and depression may be related to their conceptual similarity to established depressive dimensions. Early maladaptive schemas (EMS) are deep-seated cognitive and emotional constructs formed during childhood in response to unmet basic needs. They influence perceptions of oneself, others, and the world, and can promote depression by leading to recurring negative interpretations. People thus tend to confirm their dysfunctional beliefs, adopt avoidance or compensatory behaviors, and develop relationship difficulties that accentuate isolation. By altering emotional regulation and generating vicious circles of negative thoughts and dysfunctional behaviors, EMS contribute to the development and maintenance of depressive disorder (1, 2).

### Effects of antidepressant treatment on EMS

In our study, mean EMS scores, with the exception of the “entitlement” schema, decreased significantly after three months of antidepressant treatment.

The stability of the entitlement schema may be attributed to its frequently egosyntonic nature, as it is often perceived as an integral part of the individual’s personality rather than a symptom, it may be less susceptible to short-term mood fluctuations or pharmacological intervention alone.

Two non-mutually exclusive hypotheses may explain the broader reduction in EMS scores. First, antidepressant treatment may exert a direct effect on underlying cognitive processing. Second, the observed changes may be an indirect consequence of mood improvement, which can either positively influence self-perception and schema activation, or reflect a measurement bias inherent to self-report instruments (i.e., mood-state dependency).

Our findings are consistent with other studies reporting schema reductions after treatment. Wegener et al. examined the effect of an integrative psychodynamic inpatient therapy that did not explicitly target schemas, in a sample of 47 patients with major depression assessed with the Young Schema Questionnaire before and after treatment. Early maladaptive schemas were significantly reduced in three of the five schema domains, and stronger endorsement of schemas at baseline tended to predict symptom reduction (17). A prospective Turkish study (10) examined the effects of two months of antidepressant treatment on EMS in 40 patients with major depressive episodes. While the authors reported significant reductions in several schemas (such as abandonment, mistrust, vulnerability, failure, and self-sacrifice), a substantial number of other schemas, including entitlement, remained stable. In contrast, our study demonstrated a more generalized reduction across almost all schemas. This discrepancy might be explained by our longer treatment duration (three months versus two months), suggesting that a prolonged pharmacological intervention may be required to mobilize a broader range of cognitive structures.

However, it remains difficult to definitively determine whether these changes reflect a direct modification of underlying cognitive structures or are secondary to improvements in mood state. In support of our first hypothesis regarding a direct cognitive effect, Harmer et al. demonstrated that acute antidepressant administration can modify emotional processing biases very early in treatment, even before observable changes in mood and clinical symptoms. These early changes in emotional processing may facilitate subsequent mood improvement by enabling patients to reinterpret social and emotional information in a more positive manner. Indeed, these findings may be interpreted in light of neurocognitive models suggesting that antidepressants modify emotional bias and information processing, which could secondarily influence schema activation (18). Neuroimaging studies further suggest partially overlapping neural mechanisms between pharmacological treatments and cognitive therapies (19), reinforcing the idea of a continuum between neurobiological and cognitive models of depression.

Conversely, addressing our second hypothesis, these results must also be interpreted in light of the mood-state dependence hypothesis, according to which cognitive vulnerability remains latent and becomes activated primarily during dysphoric states. Accordingly, depressed individuals may report higher levels of maladaptive schemas when experiencing negative mood states (20). In this perspective, the reduction in EMS scores observed in our study may reflect, at least in part, an improvement in mood and a reduction in negatively biased schema activation, rather than a direct, deep restructuring of core cognitive schemas.

Overall, these findings suggest that early maladaptive schemas may exhibit a degree of cognitive plasticity in the context of depressive disorders and their treatment. This interpretation is supported by Dozois et al., who found in a randomized clinical trial that depression severity, automatic thoughts, and dysfunctional attitudes improved comparably under pharmacotherapy alone and under pharmacotherapy combined with cognitive therapy, whereas deeper changes in cognitive organization emerged only when cognitive therapy was added (21). This suggests that antidepressant treatment alone may reduce schema scores primarily through mood improvement, while a structural modification of schemas may require the addition of psychotherapy They also highlight the complexity of the relationship between mood, cognition, and therapeutic interventions. Taken together, these findings support the potential value of combining pharmacological treatment with psychotherapeutic approaches, particularly for targeting structural changes in schemas that appear more resistant to change, such as entitlement.

### Relationship between EMS and severity of depressive symptoms

In our study, no significant correlations were found between EMS scores and the severity of depressive symptoms at baseline or after three months of treatment, as measured by the Hamilton Depression Rating Scale. This finding contrasts with previous studies reporting significant associations between certain EMS and depressive symptom severity.

For instance, Renner et al. identified abandonment/instability, failure, and emotional deprivation as significant predictors of depressive symptom severity at baseline using multivariate analyses (9) (9). Schosser et al. found that higher EMS scores, especially in the schema domains of Disconnection/Rejection and Impaired Autonomy/Performance, correlated with higher depression levels at admission. Regression models explained 46% of symptom variance (20). Previous research suggests that more pronounced EMS may contribute to the development and maintenance of depressive symptoms through the activation of recurrent negative thoughts and cognitive biases, thereby amplifying emotional distress and maladaptive behaviors (22).

However, our findings may suggest that EMS are not merely reflections of current symptom severity but may represent relatively independent vulnerability factors. This dissociation could indicate that EMS operate as underlying cognitive structures that are only partially influenced by fluctuations in mood state. Alternatively, the absence of significant associations may be explained by limited statistical power or sample characteristics.

These results highlight the complexity of the relationship between cognitive vulnerability and symptom expression in depression and underscore the need for further research to clarify the temporal and causal links between EMS and depressive symptomatology.

The magnitude of improvement in depressive symptoms (ΔHDRS) was not significantly correlated with the magnitude of reduction in early maladaptive schemas (ΔEMS), either for the total EMS score or for any individual schema after correction for multiple comparisons. If schema scores merely reflected the current mood state, patients exhibiting the greatest reduction in depressive symptoms would be expected to show the greatest decrease in EMS scores. However, this pattern was not observed. These findings therefore do not support the hypothesis that the observed reductions in EMS were solely attributable to mood-state improvement or symptomatic remission. Rather, they suggest that schema changes may represent processes extending beyond the alleviation of depressive symptoms. This interpretation is consistent with the findings of Renner et al., who reported that early maladaptive schemas retained good relative stability across 16 weeks of evidence-based treatment for depression, supporting the view that EMS possess a substantial trait-like component that is not fully reducible to current mood state (9). Nevertheless, given the uncontrolled observational design of the present study, these findings should be interpreted with caution, and future controlled longitudinal studies are needed to determine whether the observed schema changes reflect genuine cognitive restructuring or other mechanisms associated with recovery from depression.

### EMS and course type of depressive disorder

We did not find any significant differences between the scores of the different EMS based on the course type of depressive disorder.

Our results are inconsistent with the data in the literature.

Özaslan et al. reported higher scores for the defectiveness/shame, abandonment/instability, and failure schemas in patients with persistent depressive disorder compared to those with recurrent depressive disorder (23).

Similarly, Chen et al. showed that several schemas, including abandonment/instability, mistrust/abuse, emotional deprivation, defectiveness/shame, social isolation, dependence/incompetence, vulnerability, enmeshment, failure, emotional inhibition, and entitlement, were significantly higher in persistent depressive disorder than in major depressive disorder or in controls (24).

These results suggest that EMS, by inducing a biased and negative perception of situations, foster the emergence of dysfunctional thoughts, maladaptive emotional responses, and ineffective behaviors. This cognitive rigidity contributes to the persistence, recurrence, and treatment resistance of depressive episodes, while increasing vulnerability to subsequent stressors and reducing the patient’s capacity to develop effective coping strategies (4).

### Strengths and limitations of the study

The primary strength of our study lies in its prospective, longitudinal design. Moreover, to our knowledge, it is the first Tunisian study to examine the relationship between depressive disorders and dysfunctional cognitive schemas. This provides valuable data from a North African population, contributing to the limited cross-cultural literature on EMS and depression. At the international level, it is also among the few studies to investigate the impact of pharmacological treatment on these early maladaptive schemas and question their stability under treatment.

However, our findings must be interpreted in light of certain methodological limitations. First, the absence of a control group prevents us from establishing definitive causal relationships. Although a significant reduction in EMS scores was observed after antidepressant treatment initiation, these changes cannot be attributed solely to the pharmacological intervention. They may also reflect the natural course of recovery from a depressive episode, nonspecific therapeutic effects associated with routine psychiatric care, statistical phenomena such as regression to the mean, or increased familiarity with the self-report questionnaire following repeated administration. Consequently, the observed changes should be interpreted as an association rather than evidence of a direct treatment effect. Future controlled studies, ideally with randomized designs and longer follow-up periods, are needed to better distinguish the specific contribution of antidepressant treatment from these potential confounding factors.

Second, while a sample size of 80 patients is robust for a clinical prospective design, it remains moderate and may limit the generalizability of our findings to the broader depressive population. Third, our reliance on self-report questionnaires renders the results susceptible to measurement bias, particularly the aforementioned mood-state dependency. Another limitation of this study is that treatment adherence was not formally assessed using a validated measure. Although most participants completed the 3-month follow-up, the potential influence of medication adherence on changes in early maladaptive schemas cannot be excluded. Finally, the specific classes and dosages of antidepressants were not strictly controlled for, which could introduce confounding variables regarding treatment efficacy.

## Conclusion

This study contributes to the ongoing debate regarding the stability of early maladaptive schemas by suggesting that they may exhibit short-term variability during depressive episodes. These findings support a more dynamic conceptualization of EMS.

Our findings have several clinical and therapeutic implications. First, they emphasize the importance of assessing maladaptive early schemas in patients with depression, in order to identify markers of cognitive vulnerability and guide appropriate therapeutic interventions. Second, the significant reduction in certain EMS following antidepressant treatment suggests that pharmacotherapy may act not only on mood but also on underlying cognitive processes.

However, some schemas, such as **“**entitlement**”**, appear more resistant to pharmacological treatment, indicating that medication alone may be insufficient to produce lasting changes in certain EMS. Nevertheless, this interpretation should be considered cautiously, as the present design does not allow differentiation between specific pharmacological effects and other factors contributing to changes over time. These results highlight the potential benefit of combining pharmacotherapy with schema-focused interventions, particularly for treatment-resistant schemas.

Finally, these observations open avenues for further research into the cognitive plasticity of EMS and the mechanisms underlying their evolution during depressive treatment. Future longitudinal controlled studies are needed to clarify whether EMS changes are specifically related to pharmacological interventions or reflect broader processes associated with clinical improvement. A better understanding of these mechanisms could enable more personalized interventions, thereby optimizing therapeutic efficacy and preventing relapse.

## Data Availability

The original contributions presented in the study are included in the article/supplementary material. Further inquiries can be directed to the corresponding author.

## Appendix 1

Box 1 Description of the young schema questionnaire (YSQ-S2). The short version of the Young Schema Questionnaire (YSQ-S2) is a self-report questionnaire consisting of 75 items and measuring 15 maladaptive early schemas 1. Emotional deprivation: past and present complaints about a lack of emotional sharing with others, lack of consideration and affection. This corresponds to items 1 to 5. 2. Abandonment/instability: This is the perception of a lack of reliability or stability in the relationship between the patient and the important people in their life. It manifests as a constant fear of being abandoned. It corresponds to items 6 to 10. 3. Mistrust/abuse: The impression that others are acting for hidden motives and seeking to cause harm. It corresponds to items 11 to 15. 4. Social isolation: loneliness, rejection by others, inability to invest in a relationship. This corresponds to items 16 to 20. 5. Defectiveness/shame: The subject judges themselves to be imperfect, inferior, bad, or incapable. This schema, manifests itself in hypersensitivity to criticism, rejection, and reprimands. It corresponds to items 21 to 25. 6. Failure: Belief that one’s level of ability and success is inferior to others. It corresponds to items 26 to 30. 7. Dependence/incompetence: This is the perception of being unable to cope with everyday responsibilities or problems without the help of others. It manifests itself in an excessive need for support and reassurance from others, passivity, or a lack of initiative. It corresponds to items 31 to 35. 8. Vulnerability: Excessive anxiety about threats or disasters that may occur at any time and that one will be unable to cope with. This corresponds to items 36 to 40. 9. Enmeshment: Inability to detach oneself from the opinions and influence of parents or partners. This corresponds to items 41 to 45. 10. Subjugation: submission to the will and desires of others, difficulty asserting oneself. This corresponds to items 46 to 50. 11. Self-sacrifice: The subject is concerned with meeting the needs of others at the expense of their own. They do this in order to spare others pain, avoid feeling guilty about being selfish, or maintain an emotional bond with someone they perceive as needy. This corresponds to items 51 to 55. 12. Emotional inhibition: inability to show and express emotions. This corresponds to items 56 to 60. 13. Unrelenting standards: desire for perfection, inability to be satisfied with one’s actions. This corresponds to items 61 to 65. 14. Entitlement: rejection of rules and conventions, delusions of grandeur. This corresponds to items 66 to 70. 15. Insufficient self-control: lack of discipline and willpower to complete tasks, refusal to act against one’s will. This corresponds to items 71 to 75. Each item is rated on a scale of 1 to 6 (1 = This has never been true of me; 2 = This has been true of me at some point in my life; 3 = This is currently true of me; 4 = fairly true for me; 5 = very true for me; 6 = describes me perfectly). Each subject is given an overall schema score and a specific score for each dimension. The specific scores for each of the fifteen schemas can range from 5 to 30. The overall schema scores can range from 75 to 450.
